# Adaptable Neighbours: Movement Patterns of GPS-Collared Leopards in Human Dominated Landscapes in India

**DOI:** 10.1371/journal.pone.0112044

**Published:** 2014-11-12

**Authors:** Morten Odden, Vidya Athreya, Sandeep Rattan, John D. C. Linnell

**Affiliations:** 1 Faculty of Applied Ecology and Agricultural Sciences, Campus Evenstad, Hedmark University College, Koppang, Norway; 2 Wildlife Conservation Society – India & Centre for Wildlife Studies, Banashankari, Bangalore, Karnataka, India; 3 Wildlife Wing, Himachal Pradesh Forest Department, Talland, Shimla, Himachal Pradesh, India; 4 Norwegian Institute for Nature Research, Trondheim, Norway; University of KwaZulu-Natal, South Africa

## Abstract

Understanding the nature of the interactions between humans and wildlife is of vital importance for conflict mitigation. We equipped five leopards with GPS-collars in Maharashtra (4) and Himachal Pradesh (1), India, to study movement patterns in human-dominated landscapes outside protected areas. An adult male and an adult female were both translocated 52 km, and exhibited extensive, and directional, post release movements (straight line movements: male  = 89 km in 37 days, female  = 45 km in 5 months), until they settled in home ranges of 42 km^2^ (male) and 65 km^2^ (female). The three other leopards, two adult females and a young male were released close to their capture sites and used small home ranges of 8 km^2^ (male), 11 km^2^ and 15 km^2^ (females). Movement patterns were markedly nocturnal, with hourly step lengths averaging 339±9.5 m (SE) during night and 60±4.1 m during day, and night locations were significantly closer to human settlements than day locations. However, more nocturnal movements were observed among those three living in the areas with high human population densities. These visited houses regularly at nighttime (20% of locations <25 m from houses), but rarely during day (<1%). One leopard living in a sparsely populated area avoided human settlements both day and night. The small home ranges of the leopards indicate that anthropogenic food resources may be plentiful although wild prey is absent. The study provides clear insights into the ability of leopards to live and move in landscapes that are extremely modified by human activity.

## Introduction

In Europe and North America, it has long been understood that the conservation of highly mobile wildlife species, especially the large carnivores, will require substantial populations to range across multi-use landscapes outside protected areas that are simply not large enough to support viable populations. Under supportive legislation and the recovery of forest habitats and wild prey, both continents have seen dramatic recoveries of species as iconic as wolves *Canis lupus*, mountain lions *Puma concolor*, brown bears *Ursus arctos* and Eurasian lynx *Lynx lynx* [1], [2], [3]. Conservationists in tropical countries have been much slower to see the conservation value of multi-use landscapes. However, a series of papers have recently focused on the potential for secondary forests and agri-forest systems to house significant biodiversity[4]. There is also an emerging body of evidence showing that large carnivores can also thrive in multi-use landscapes in tropical countries[5], [6].

Leopards *Panthera pardus* are among the most successful of the large tropical carnivores in terms of abundance and geographic distribution. The broadness of their ecological niche is reflected in their presence in widely variable environments, ranging from open and semi-arid deserts, through savannahs to tropical forests [7]. Nonetheless, leopards were re-categorized from Least Concern to Near Threatened in the 2008 revision of the IUCN red lists [8]. The reason behind the changed protection status was a perceived decrease in abundance and distribution in parts of their range; patterns that were attributed to direct human persecution and destruction of habitats. Leopards are, however, sympatric with several vulnerable and endangered large felids, i.e. tigers *Panthera tigris*, lions *Panthera leo*, and cheetahs *Acinonyx jubatus*, that are far more vulnerable to human impacts. Leopards appear to be better able to tolerate humans, and their foraging habits are highly flexible [9], thus allowing leopards to persist in areas of low wild prey availability (by consuming domestic animals) and high human pressure [10]. The adaptability of leopards is therefore coupled with a high potential for conflicts with humans, a problem that is currently regarded as one of the greatest threats to the conservation of large carnivores worldwide [11], [12]. Hence, it is important to improve our understanding of how leopards interact with people, in order to minimise the inevitable conflicts that follow their sympatry with humans.

Few ecological studies of leopards have been conducted in India, and even fewer outside protected areas [13], rendering basic ecological knowledge either lacking or limited in several aspects that are relevant for leopard conservation and management. The need for ecological knowledge and its association with practical conflict resolution is evident in India, where leopards are found in many areas with high human population densities, and are involved in more conflicts than any other large carnivore in the country [14], [15]. Present conflict management in India is generally reactive; based on *ex post facto* compensation of damage to livestock and humans, and the haphazard capture and/or translocation of individuals that are believed to be prone to problematic behavior [15]. However, a recent study showed that large-scale translocation of leopards increased the subsequent level of conflicts [15]. The authors suggested that increasing frequencies of attacks on humans following translocation were potentially caused by behavioural changes following stress and aggression induced during the translocation procedure and accidental encounters with humans during movements through unfamiliar terrain at the release site. These findings suggest that conflict mitigation requires more focus on pro-active mitigation measures aimed at facilitating coexistence. One of the misconceptions that have underpinned the reactive management in India is the idea that leopards in human-dominated landscapes are not resident; instead it is often claimed that they are transient ("stray") dispersers from protected areas in need of "assistance". Addressing these issues requires detailed knowledge of how leopards actually use human-dominated landscapes, i.e. studies of space use, foraging behavior and their interactions with people. In the present study, we seek to answer these questions through a detailed account of spatio-temporal patterns of leopard range use and movements outside of protected areas in India using GPS telemetry. We provide data on home range sizes and investigate leopard movements in relation to the distribution of human settlements. Furthermore, we compare movements of leopards that were translocated long distances (>50 km) with those of leopards released near their capture sites (<10 km). Although our sample size is low (n = 5) this is the first GPS based study of leopards ever conducted in India, and no leopard study has ever been conducted in such a human dominated landscape.

### Ethics statement

This study used data from GPS collared leopards which were captured, collared and monitored with the permission of the Ministry of Environment and Forests, New Delhi F. No. 1-4/2007 WL-1 dated 8 April 2008 and the Forest Department of Himachal Pradesh, letter number WL/Study-Research/37-21 dated 22 September 2010. The leopard is protected by the Indian law under the Wildlife Protection Act and the Forest Department is the administrative body that is responsible for wildlife welfare under Indian law. There was no other animal ethic committee legally required to approve this work. Norwegian research on animals is regulated by the “Law on animal welfare (LOV-2009-06-19-97) (http://lovdata.no/dokument/NL/lov/2009-06-19-97?q= dyrevelferdsloven). Paragraph 2 on the area of operation explicitly states that the law only applies to “Norwegian land area, territorial waters, the Norwegian economic zone, Norwegian ships and airplanes, Norwegian installations on the continental shelf, and Svalbard, Jan Mayen and other islands [Antarctic possessions]“, so that no formal approval was needed from Norwegian sources for the Norwegian members of the team to take part. We made sure that all attempts were made to reduce stress to the animal before, during and after collaring until the time of release. In all cases, a trained veterinarian was present to carry out the tranquilisations. Furthermore, Forest Department staff were part of all collaring operations.

### Study Areas and Methods

**Figure 1:**
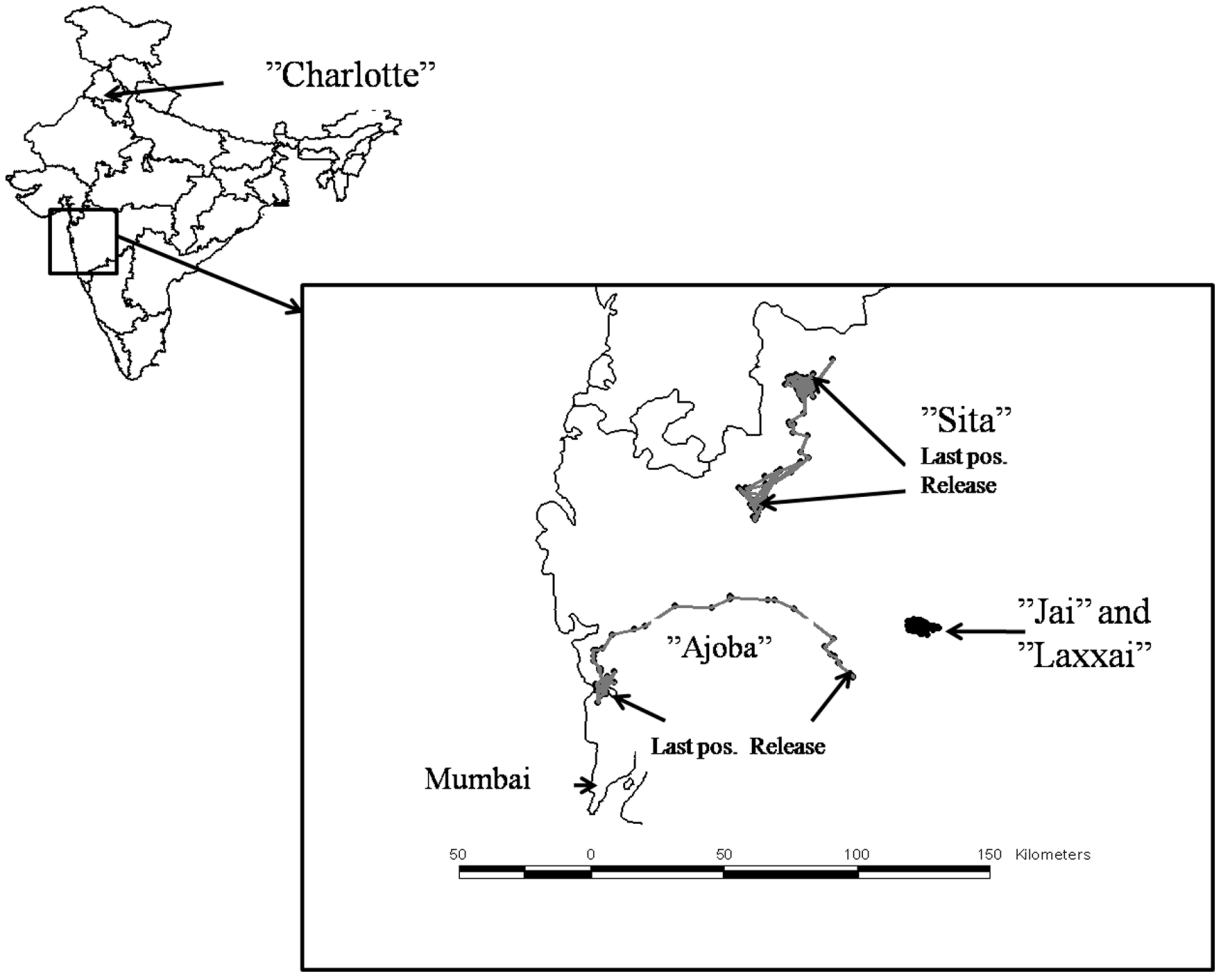
Overview of locations of five GPS-collared leopards captured in the states of Maharashtra and Himachal Pradesh, India.

A total of five leopards were equipped with GPS-collars in this study; four were captured in the state of Maharashtra and one was captured in the state of Himachal Pradesh (Figure 1). Two of the Maharashtrian leopards, “Jai” (male) and “Laxxai” (female), were captured within an area of an ongoing intensive human-leopard conflict study (see Athreya et al. [10], for a detailed description of the area), in a densely populated irrigated valley in Akole Tahasil (sub district) located in the western edge of Ahmednagar district (19.576959 N 73.937123 E to 19.460715 N 74.089954 E). Akole Tahasil contains 191 villages (as per 2001 census) and covers an area of 1505 km^2^ with an overall human population density of 177 per km^2^, although the operational density in the irrigated valleys where the leopard study was conducted was over double this value. Most land in the valley bottoms is used for the intensive cultivation of irrigated crops such as sugar cane, while the surrounding dry hills are heavily grazed by livestock or used for the seasonal cultivation of other rain-fed crops that do not need irrigation.

In addition, we collared two leopards that were captured and translocated by the Forest Department in Maharashtra. These were “Ajoba” who had fallen in a well in Parner (Ahmednagar district), and “Sita” who had run into a house in Surghana (Nashik district) (Figure 1). Both the districts of Ahmendnagar and Nashik have relatively high human population densities of 266 and 393 per km^2^, respectively. However, the Surgana sub-district where the leopard “Sita” was captured is a forested region (not part of a protected area), with a predominantly tribal population who mainly cultivate paddy. The last leopard, “Charlotte”, was captured in a box trap set up about 4 km from the capital of Shimla (Shimla Rural district, Himachal Pradesh), in an area with a human population density of 159 per km^2^. The natural habitat of the area comprises of highly inaccessible mountainous terrain with altitude varying from 1375 to 2050 m.a.s.l., housing wild populations of pheasants, barking deer, gorals and wild boars etc. The regular movements of the leopard were confined near human habitations. Latitudes and longitudes of all capture- and release locations are provided in Table 1.

**Table 1:** Details of the captures and releases, and home range sizes (during periods of stability), of five GPS- collared leopards outside protected areas in India.

Name	Sex and age	Weight (kg)	Dates		Location coordinates		Home range sizes (km^2^)		
			Capture	Last pos.	Capture	Release	95% MCP	95% kernel	75% kernel
Ajoba	AM	63	28.04.2009	17.07.2009	19.1021N	19.3350N	45.36	42.01	11.87
					74.2194E	73.7845E			
Jai	YM	45	01.05.2009	16.12.2009	19.5415N	19.5339N	10.87	8.35	2.64
					73.9907E	74.0553E			
Laxxai	AF	36	04.05.2009	04.05.2010	19.5489N	19.5339N	10.91	10.91	2.23
					73.9636E	74.0553E			
Sita	AF	35	26.05.2009	27.05.2010	20.4064N	19.9694N	68.66	64.80	18.34
					73.6051E	73.4312E			
Charlotte	AF	35	16.09.2010	11.04.2011	31.1482N	31.1019N	8.43	14.88	8.15
					77.1595E	77.1435E			

A =  adult (>3 years), Y =  young (1–2 years), M =  male, F =  Female.

The five leopards that were captured during this study were fitted with Vectronics (Vectronics Aerospace GPS Plus I) collars with Lotek 52 weeks pre-programmed drop-offs. The GPS locations were transmitted over the GSM network. The animals were trapped in box traps and tranquilised with a blowpipe using ketamine (∼5 mg/kg) and xylazine (∼2 mg/kg) for the collaring procedure. Yohimbine (0.14–0.17 mg/kg) was used for hastening the reversal when required. In order to reduce disturbance and stress, silence was maintained prior and during the immobilization procedure and the cages were covered on all sides. Only a veterinarian and one more staff member approached the animals for tranquilisation. Once the leopards were tranquilised, care was taken to keep the tongue outside the mouth and to keep the head straightened to respiration. Temperature, respiration, and heart rate were recorded every 10–15 minutes. We determined the age of the leopards based on tooth wear [16] and other phenotypic characteristics [17]. Accordingly, one leopard, “Jai”, was classified as young (1–2 years), whereas the other four leopards were classified as adults (>3 years). See Deka et al. [18] for further descriptions of capture procedures.

**Figure 2:**
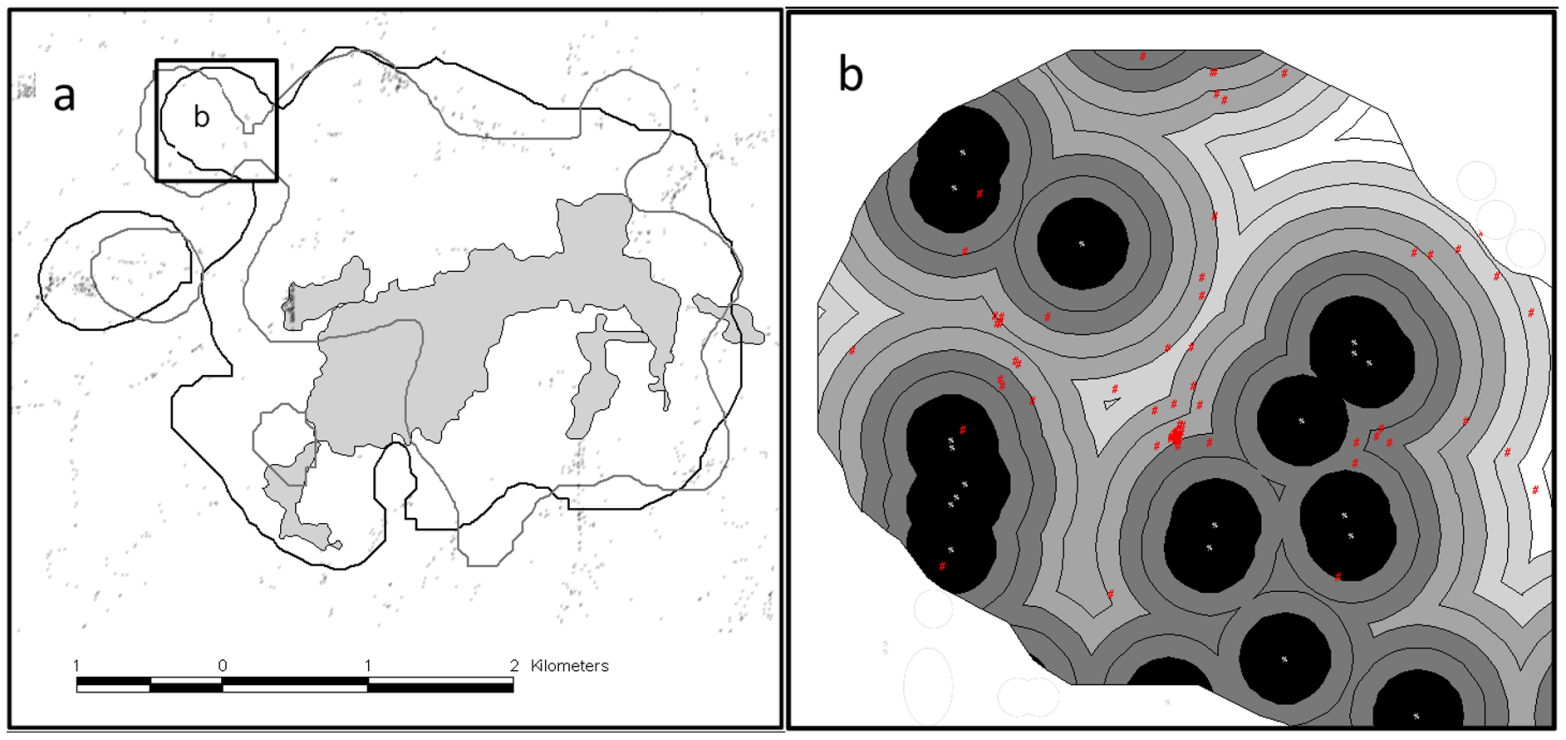
95% Fixed kernel home ranges of two leopards in Maharashtra India. (a) Home range borders of a subadult male (grey line) and an adult female (black line) leopard living around Akole village (grey polygon). Individual houses are indicated by grey dots. (b): A subset of the home range of the female leopard showing individual houses (white dots) with 25 m buffers that were used to calculate distances from GPS-locations to residential houses and to estimate the expected distances to houses if the distribution of GPS fixes was random within each leopard home range. Red dots represent GPS fixes of the leopard.



The location data were collected following two sampling schedules. For three weeks of each month, locations were taken once every 3 hours, and for one week a month, intensive locations were taken every hour. We delineated home range borders (animal movement extension in ArcView 3.3) with a 95% fixed kernel estimator (least squares cross validation and individual smoothing factors; [19], Figure 2a). Minimum Convex Polygons (MCP95, [20]) excluding 5% of the locations furthest away from the harmonic mean center were also calculated as range estimators. In addition, we used a 75% fixed kernel estimator to represent core areas. These two latter values are presented in Table 1. All other values in the text refer to 95% kernel estimators. For home ranges and core areas we used night time positions with a minimum of 24 h interfix intervals to reduce auto-correlation. By excluding daytime positions we ensured that home ranges were delineated mainly based on locations from periods of activity, i.e. from periods when territorial borders were patrolled. Furthermore, daytime positions were more clustered than nighttime positions and therefore less suitable for accurate determination of home range borders using kernel analysis [21]. Hourly positions from the intensive periods were used for analyses of movement distances during the diel cycle. In order to investigate movement patterns and range use in relation to the distribution of human settlements we mapped and digitized (from Google Earth and ground based surveys) all residential houses within the home ranges of four leopards, and measured the distances from all GPS fixes to the nearest house. We measured distances to houses by placing a series of circular buffers with borders in intervals of 25 m around each house, i.e. at 25 m, 50 m, 75 m etc., until the whole home range was covered with buffers. We assigned a value of distance to the nearest house by overlaying the leopard GPS locations on the map with buffers (Figure 2b). Hereafter these values are referred to as the “observed” distances to the nearest residential houses, whereas “expected” distance are the average minimum distances to residential houses at any location within the borders of each of the 95% kernel home ranges, i.e. the expected distance to the nearest house if locations were randomly distributed. Hence, one “expected” distance value was assigned to each animal and patterns of “attraction” or “avoidance” of residential houses was quantified by subtracting the “expected” distances from the “observed” distances (i.e. observed – expected distances). In order to calculate the “expected” average minimum distances to the nearest house within each home range we divided the product of the buffer areas and the distance to houses with the total area of the home range (equation 1).

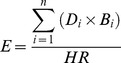
(1)Where E =  expected average minimum distance to residential houses within a home range, D =  distance from a buffer to the nearest residential house (i.e. 25m, 50m, 75m etc), B =  the area of a buffer with a given distance (D) to the nearest residential house, n =  the number of buffers, HR =  the size of the 95% fixed kernel home range.

In the analyses of movement patterns we excluded data from the translocated adult male leopard (“Ajoba”) because his collar stopped functioning after a relatively short time period. Hence, we included data from the two leopards captured near Akole town (“Jai” and “Lakshai”), the leopard captured in Himachal Pradesh (“Charlotte”) and the female leopard captured in the Nashik area (“Sita”). For the latter we only included data from the period after she had returned to the vicinity of her capture site.

We used Generalised Linear Models (GLM) for analysing the movement data. Response variables were (i) hourly step length, (ii) distance to the nearest residential house and (iii) the difference between observed distances to houses and the expected distances with a random distribution of fixes within the leopard home ranges (see above). The latter response variable was termed the “Standardised distance to the nearest house”, and obtained positive values if GPS locations were further away than expected from a random distribution, i.e. “avoidance”, and negative values if they were closer, i.e. “attraction”. In all the response variables, we averaged the values over 12 hour periods in order to reduce autocorrelation. Hence, the response variables (i, ii and iii) represent average values for each day (6am–6pm) and night (6pm–6am). Explanatory variables were leopard ID and time of day (day or night: DN). Thus, for each response variable, we compared five different models: M1 = ID, M2 = DN, M3 = ID+DN, M4 = ID+DN+ID*DN, M5 =  Null model with only intercept. We ranked and evaluated the models based on Akaike Information criterion (AIC) and Akaike weight (w) values [22].

## Results

Two of the collared leopards, an adult male (“Ajoba”) and an adult female (“Sita”) were both translocated and released 52 km from their sites of capture (Figure 1, Table 1). Following their release, both leopards moved long distances, and the last locations of "Ajoba" and "Sita" were 89 and 45 km from their release sites, respectively. “Ajoba” moved rapidly westward and reached the outskirts of Mumbai city after 37 days (Figure 1), where he established a 42 km^2^ home range (n = 42 GPS locations). He used this area until his collar stopped functioning 42 days later. He is likely to have resided there as he was found dead after a road accident 2.5 years later in the same region. None of his movements were orientated towards his capture location. “Sita” moved slowly north from her release site towards the site of her capture. After 5 months she reappeared near the capture site and established a home range of 65 km^2^ (n = 213) where she remained until the collar dropped off 7 months later.

The three other leopards, a young male “Jai”, and the adult females “Lakshai” and “Charlotte”, were released in the immediate vicinity (<10 km) of their capture sites (Table 1). All these leopards moved towards their respective capture sites immediately after release. Charlotte used a home range area of 15 km^2^ during 7 months of GPS tracking (n = 207). Jai and Lakshai used overlapping home ranges of 8 (n = 225) and 11 (n = 364) km^2^ that were situated within and around the borders of Akole town in Maharashtra (Figure 2a). Jai was recaptured after entering a house in December 2009 after which his collar was removed. Lakshai's collar dropped off one year after capture, as scheduled. All study leopards occupied small, discrete, and very stable home ranges implying that they were resident in their ranges. Both "Lakshai" and "Sita" raised cubs during the study period.

**Figure 3:**
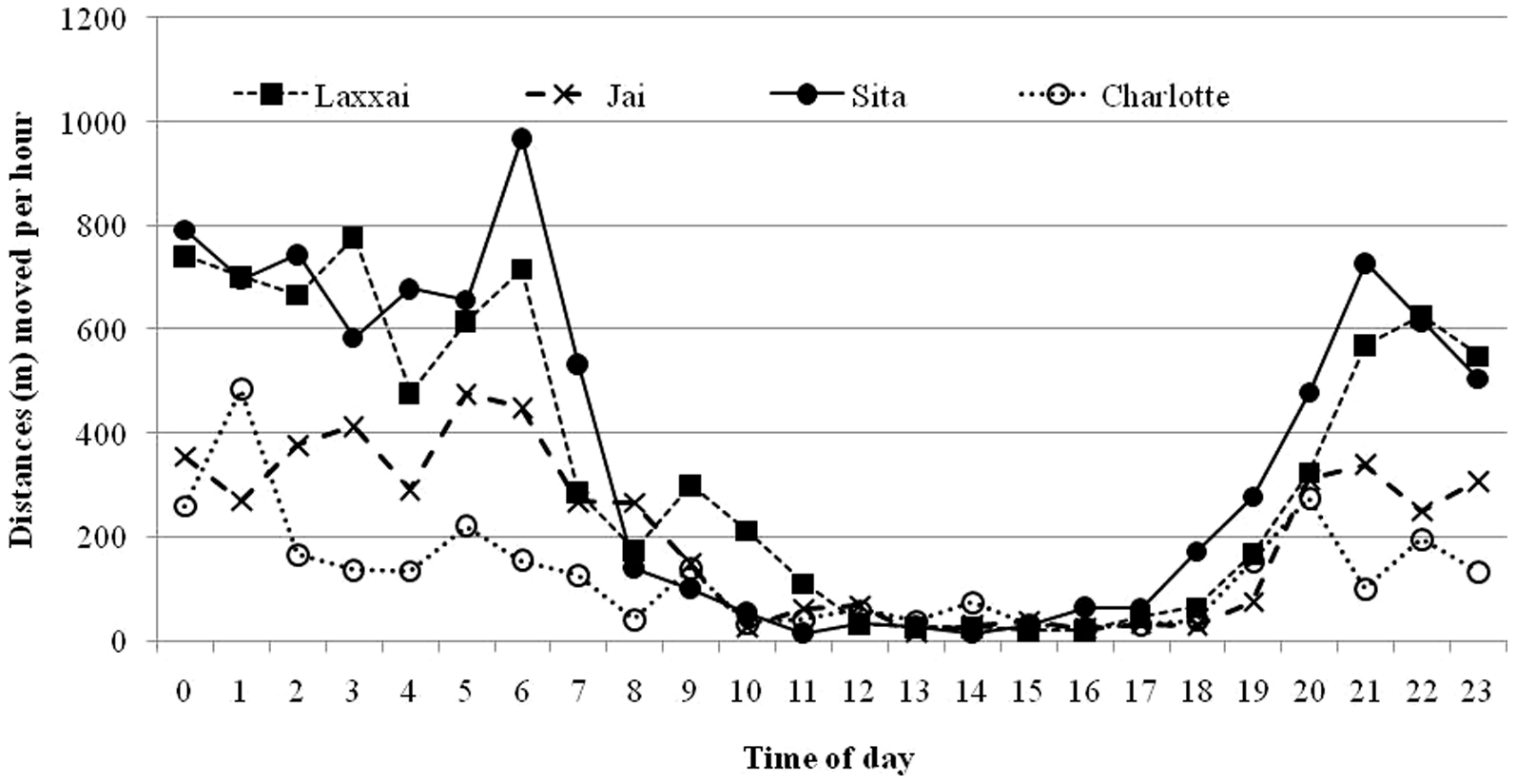
Distances moved per hour during 24h intensive GPS tracking of three adult female leopards (Laxxai, Sita Charlotte) and one subadult male (Jai) outside protected areas in India.

The analysis of the distances moved during the diel cycle revealed a pronounced nocturnal behavior among all the leopards (Figure 3, Table 2). The average hourly step length was 339±9.5 m during night and 60±4.1 m during day, and time of day (day vs. night) was a factor included in the highest ranking model of hourly movement distances (Table 3 and 4). This model also included animal ID, indicating differences among the leopards in hourly movement distances, and an interaction between ID and time of day implying that the relative distribution of movement between day and night differed among the leopards. The two leopards living in the most human dominated landscape around Akole town, “Jai” and “Lakshai”, moved 5.3 and 7.7 times longer during night than during day, respectively, whereas the other two leopards (“Sita” and “Charlotte”) moved approximately 3.5 longer during night than during day (Table 2).

**Table 2:** Average (±SE) hourly step lengths and distances to the nearest residential houses of four GPS-collared leopards outside protected areas in the states of Maharashtra and Uttar Pradesh, India.

ID	Hourly step lengths (m)		Distances to houses (m)		Standardised distances to houses (m)	
	Night (n)	Day (n)	Night (n)	Day (n)	Night (n)	Day (n)
Charlotte	195.2±17.7 (20)	55.3±8.5 (20)	152.4±4.8 (204)	245.6±5.0 (204)	−27.6±4.8 (204)	65.6±5.0 (204)
Jai	275.6±13.4 (60)	51.6±6.8 (60)	87.9±3.2 (218)	154.6±4.6 (218)	0.9±3.2 (218)	67.6±4.6 (218)
Laxxai	369.7±15.0 (76)	48.1±4.8 (76)	110.2±2.6 (357)	161.8±2.7 (357)	18.2±2.6 (357)	69.8±2.7 (357)
Sita	416.7±56.4 (27)	116.3±21.1 (27)	1440.4±41.0 (203)	1929.8±35.6 (203)	309.4±41.0 (203)	798.84±35.6 (203)

The “standardised distances to houses” is the difference between observed distances between leopard locations and houses and the expected distances to houses if GPS locations were randomly distributed within the leopard home ranges. The latter variable attains positive values if GPS locations are further away from houses than expected from a random distribution (i.e. avoidance of houses), and negative values if locations were closer than expected (i.e. “attraction”).

**Table 3:** Summary of Generalised Linear Models of movements and location distribution of four GPS-collared leopards outside protected areas in India.

		Hourly step length		Distance to house		Standardised distance to house	
Model	Terms	ΔAIC	W	ΔAIC	W	ΔAIC	W
M1	ID	169.24	<0.01	66.30	<0.01	169.92	<0.01
M2	DN	23.66	<0.01	985.52	<0.01	490.95	<0.01
M3	ID + DN	7.66	0.02	20.42	<0.01	44.40	<0.01
M4	ID + DN + ID*DN	**0.00**	**0.98**	**0.00**	**1.00**	**0.00**	**1,00**
M5	NULL	179.02	<0.01	1013.03	<0.01	591.18	<0.01

The response variable “Hourly step length” denotes the linear distance between GPS locations with a time interval of one hour. The variable “Distance to house” is the linear distance between GPS locations and the nearest residential house, whereas the “Standardised distance to house” is the difference between observed distances between leopard locations and houses and the expected distances to houses if GPS locations were randomly distributed within the leopard home ranges. The latter variable attains positive values if GPS locations are further away from houses than expected from a random distribution (i.e. avoidance of houses). Explanatory variables are ID (animal ID) and DN (day or night position). NULL  =  null model with no explanatory variables, W =  Akaike weight.

**Table 4:** Parameter estimates and test statistics of the highest ranking Generalised Linear Models (lowest AIC-values) of movements and location distribution of four GPS-collared leopards outside protected areas in India.

Response variable	Predictor variable	Estimate	SE	t-value	P
Hourly step length	Intercept	221.17	90.09	2.455	0.015
	ID	140.86	32.86	4.287	<0.001
	DN	−95.52	56.98	−1.676	0.095
	ID*DN	−63.7	20.78	−3.065	0.002
Distance to house	Intercept	−413.22	100.92	−4.095	<0.001
	ID	246.73	36.4	6.778	<0.001
	DN	−112.7	63.97	−1.762	0.0783
	ID*DN	109.37	23.06	4.744	<0.001
Standardised distance to house	Intercept	−65.02	57.37	−1.133	0.257
	ID	−11.49	20.7	−0.555	0.579
	DN	−116.91	36.37	−3.215	0.001
	ID*DN	107.62	13.11	8.211	<0.001

See table 3 for descriptions of response and predictor variables.

**Figure 4:**
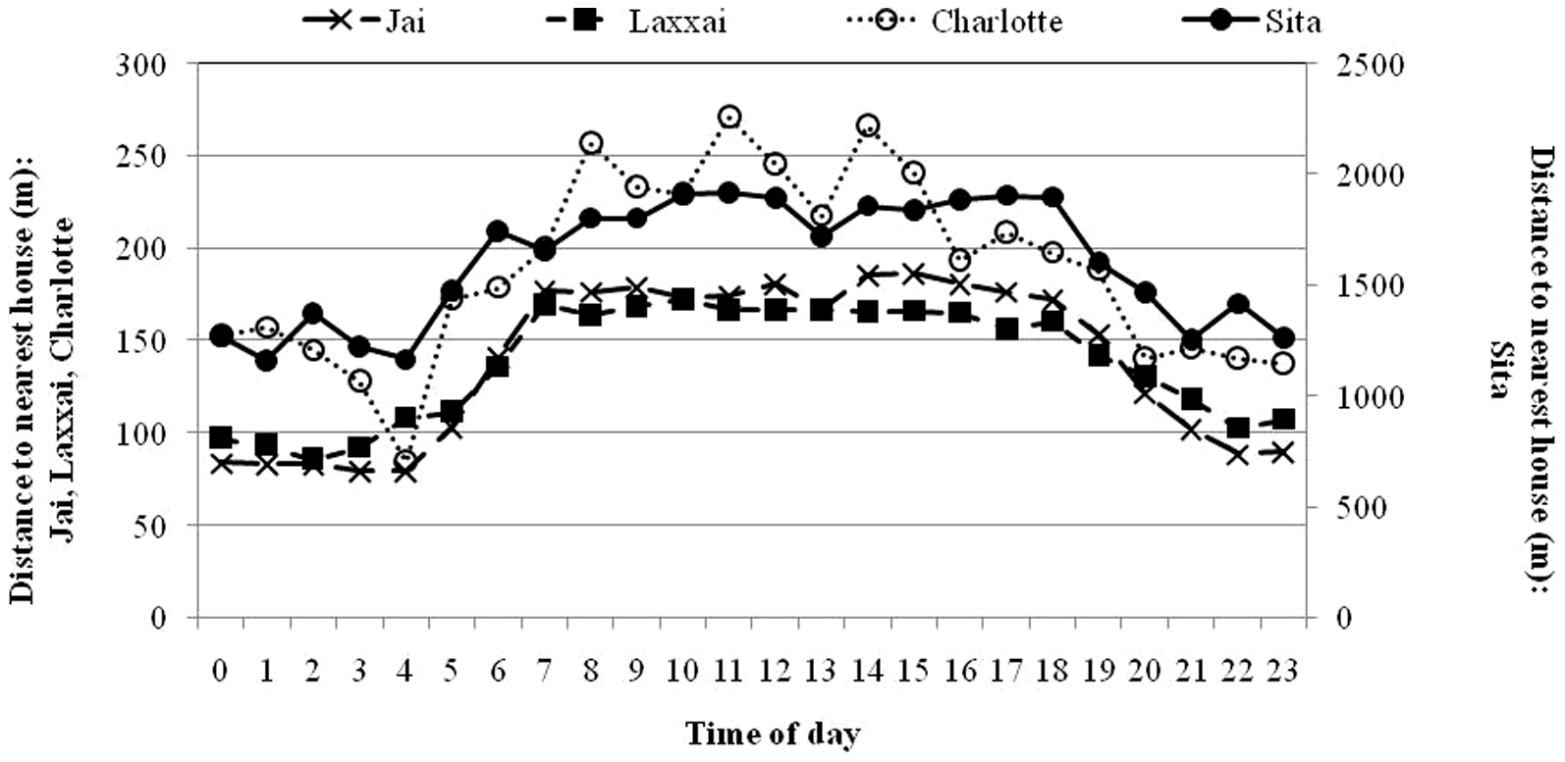
Distance to the nearest residential house in relation to time of day of three adult female leopards (Laxxai, Sita and Charlotte) and one subadult male (Jai) outside protected areas in India.

**Figure 5:**
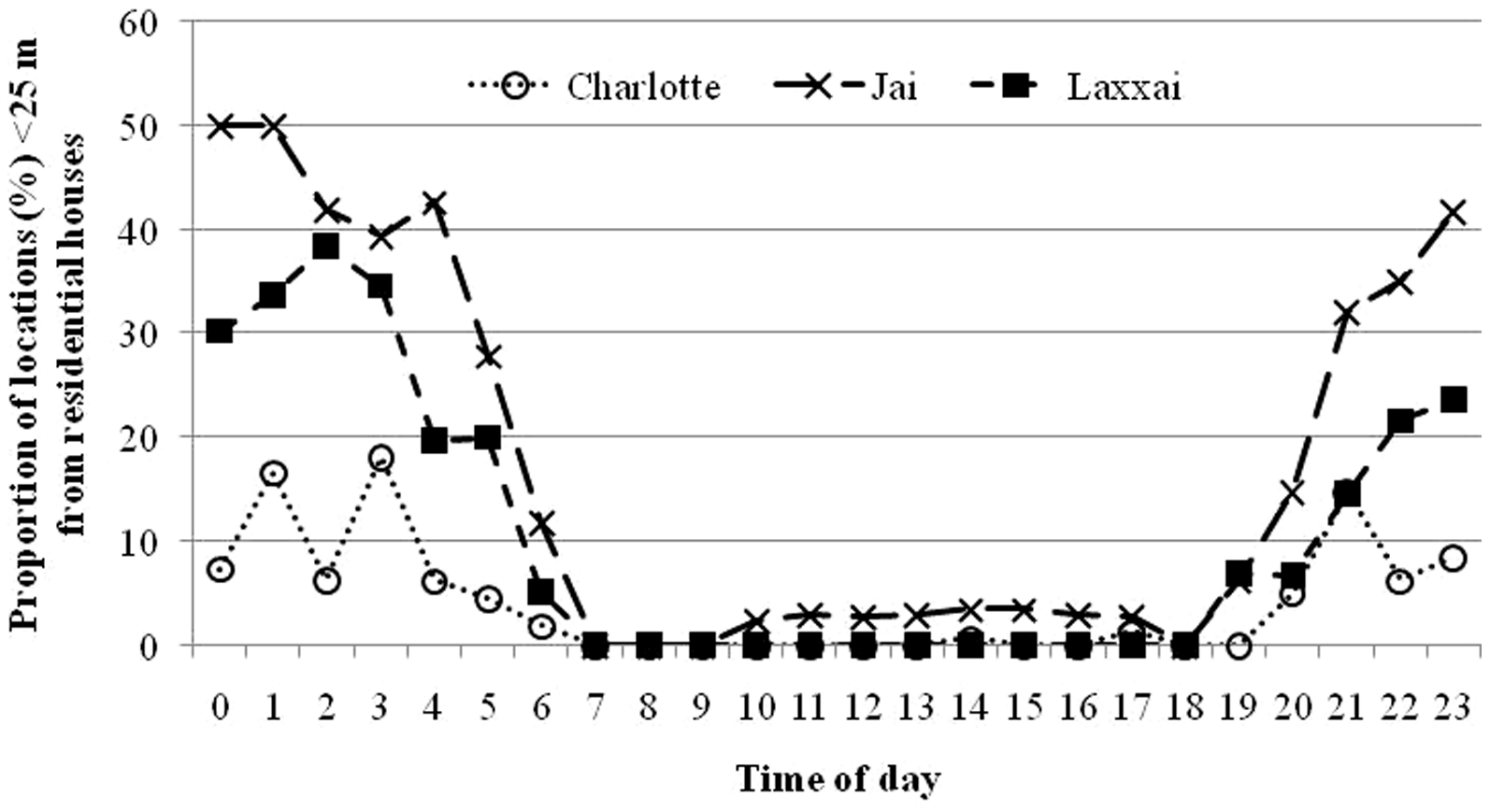
Proportions of locations of three GPS collared leopards <25 m from houses, in relation to time of day, for three leopards outside protected areas in India.

A similar pattern was revealed in the analysis of distance to nearest house. The highest ranking model of distances between leopard GPS-locations and houses included animal ID, time of day (day or night) and the interaction between these terms (Table 3 and 4). The leopards generally moved closer to houses during night, but there were marked individual differences both in average distances and their relative distribution between day and night (Figure 4). The adult female “Sita”, lived in a sparsely populated area (0.2 houses per km^2^), and her day locations were only 34% more distant from a house than at night. She was very rarely located in the immediate vicinity of houses, i.e. less than 1% of the locations were closer than 25 m. The other three leopards lived in much more densely populated areas and the difference with respect to time of day was more marked, i.e. distances to houses were more than 50% longer during day than during night. Furthermore, they were all frequently located closer than 25 m from houses at night, but very rarely during day (Figure 5).

The most parsimonious GLM model of standardized distances to the nearest house (i.e. observed – expected distances) revealed that avoidance of human settlements depended on time of day, leopard ID and the interaction between these terms (Table 3 and 4). In general, all the leopards exhibited an avoidance of houses during day, as GPS fixes were consistently further from houses than expected from a random distribution (Table 2). However, differences between the individuals were apparent during nighttime. “Sita”, living in a sparsely populated area with an expected distance of 1131 m to the nearest house within her home range, exhibited a more pronounced avoidance of houses at night than the other leopards (Table 2). The density of houses was far higher within the ranges of “Jai”, “Laxxai”, and “Charlotte”, i.e. with expected distances to the nearest house of 87, 92 and 180 m, respectively. Their standardized distances to houses were close to zero at night, thereby revealing a pattern resembling a random distribution of GPS fixes with respect to human settlements at this time (Table 2).

## Discussion

Although our earlier work in the same landscape has documented a high density of leopards [10], information from the collared animals reveals how constantly, and closely, they live in proximity to humans. The GPS collared leopards that were released close to their capture sites (<10 km) were present in the same areas until the end of the monitoring period whereas the two leopards that had been translocated far from their capture sites (ca 50 km) both moved long distances following their release, i.e. 89 and 45 km from the positions of release to the last location. These results are consistent with several previous telemetry studies of large carnivores showing that wide-ranging post-release movements are common among translocated animals (reviewed by Rogers [23] and Linnell et al. [24]).

In our study, only one of the two translocated individuals, an adult female, returned to the capture site. For unknown reasons, the other translocated leopard, an adult male, moved rapidly away from the release site, but not in the direction of his capture location. Despite being released in forested patches, both translocated leopards moved through very human dominated landscapes (including industrial and suburban areas in the case of Ajoba) in their post release wanderings. These movements indicate that the potential benefits from using translocation as a management strategy to resolve leopard conflicts with humans are very limited. It appears that relocations of so called problem individuals may either have only short-term local effects, may simply move the conflict to another area, or in the worst case scenario, increase the level of conflict [15].

Intrasexual territoriality is a general feature in large felid social organization, and the sizes of territories typically vary with respect to the density and spatial distribution of prey [25]. Interestingly, the smallest home ranges of the leopards in our study were of those individuals occupying the areas of highest human population density, i.e., “Jai”, “Lakshai” and “Charlotte”. The sizes of their ranges are among the smallest ever recorded in any leopard study, only comparable to estimates from highly productive protected areas with a high density and diversity of wild prey (reviewed by Odden and Wegge [26]). The two leopards, “Jai” and “Lakshai”, were captured in an area of an ongoing intensive study of human leopard interactions, where population densities of humans and leopards were>30000 and 5 per 100 km^2^, respectively [10]. The area is devoid of wild ungulate prey species, and the diet of leopards in the area consists mainly of domestic animals, i.e. ca 87%, especially dogs [27]. The high leopard population density and the small home ranges are both indicators of an area rich in resources, although in this case they were mainly of anthropogenic origin (livestock and pets).

The movement patterns of the leopards revealed a pronounced nocturnal behaviour and during this time, the leopards moved closer to human settlements than during daytime. However, despite these similarities among the leopards, there were marked individual differences in their average distances to houses. The adult female “Sita”, whose home range was relatively large and situated in an area with quite low human population density, stayed further from human settlements then the other leopards, and she was very rarely within the immediate vicinity (<25 m) of houses. This leopard exhibited avoidance of houses especially during day, but also at night. In contrast, “Jai”, “Laxxai” and “Charlotte”, spent a large proportion of their night-time activity periods moving very near houses (Figure 5), and avoidance of human settlements was evident mainly during daytime. Being to a large extent dependent on resources provided by humans, these leopards simply followed the distribution of their main prey, i.e. domestic animals, which are kept within or near human residencies at night. During the day when human activity was the highest, the leopards restricted their movements to areas further away from houses, although the high human density implied that there were no refuges of any considerable distance from houses. Although we did not collect systematic data on habitat use, it appeared that areas of high crops, such as sugar cane, or patches of scrub provided day-time cover. In conclusion, our results imply that temporal patterns in avoidance of humans were most pronounced in highly human-modified landscapes. However, it is important to keep in mind that more research on this topic is needed due to the limited sample size of our study.

This study exemplifies that the leopard is a highly adaptable species with an excellent ability to utilise whatever resources are available in human dominated environments. The spatiotemporal patterns of movements reveal that the leopards were able to live in an incredible degree of proximity to humans. Yet within the constraints of the area they appear to have adopted a strategy of minimising direct contact with humans to the greatest possible extent while simultaneously being dependent on domestic animals for food. To a degree, this pattern concurs with a recent study showing that tigers outside Chitwan National Park, Nepal, responded with a temporal displacement of activity as a response to human disturbance [28]. The authors of that study suggested that tiger coexistence with humans has been facilitated by a high tolerance among local people, and management actions aimed at increasing tiger prey density (e.g. banning of livestock grazing) and attempts to control poaching.

India has strict laws that prevent the killing of large felids, even after livestock are killed. Furthermore, the rural people are also much more tolerant than seen in many parts of the world and accept the presence of these species in their landscapes [13]. This tolerance is not restricted only to leopards, but also includes wolves [29], Asiatic lions [30], tigers [28] and a wide suite of smaller carnivores that occur in human-dominated landscapes in India [10]. None of the radio-collared leopards were involved in serious conflict (purposeful attacks on humans) despite having an enormous potential for such encounters. On many occasions, the collared leopards were seen by people and were sometimes even chased by people, yet no fatal attacks occurred. Perhaps this is because of the adaptability of the species to living in what could otherwise be a potentially high-risk situation because high conflict would also imply high levels of retaliation. This aspect of large felid behaviour has not been studied and needs greater attention.

The low natural prey density that follows a high human pressure on the environment implies that a certain degree of conflict is inevitable due to a lack of alternatives to domestic animals for food. Hence, if leopards are to be conserved in human dominated landscapes, it is of vital importance to evaluate and use effective conflict mitigation measures in order to maintain or increase tolerance and limit negative impacts on local people. Our results have shown that a viable natural prey base is apparently not always a prerequisite for sustaining leopards in an area, but it may affect frequencies of livestock depredation events [31].

Although the sample size in this study was rather small, it still represents a considerable addition to the published data on leopard movements which is very sparse (but see [26], [32], [33], [34], [35], [36], [37]). It also provides some very important behavioural insights that are relevant for policy. The movement of the leopards translocated over long distances supports data obtained from other studies of micro-chip tagged leopards [38] and strengthens the arguments against using translocation as a management strategy [15]. The demonstration of the manner in which the resident leopards lived and moved in the immediate proximity to humans reveals the incredible plasticity of this species' ecology and their tolerance for anthropogenic landscapes. It also confirms that the leopards living in this landscape also include resident and reproductive animals, and not only transient dispersers. Such demonstrations are very important in creating an awareness of the need for a proactive policy for leopard management that accepts that leopards are living, and will continue to live, in these landscapes. Finally, the study reveals just how adaptable some wildlife species are at occupying human-dominated tropical landscapes. The good news for wildlife is that it opens up huge new areas as potential arenas for conservation. The challenge for the management agencies that until now have focused their work on policing protected areas is that they have to work over much wider landscapes in ways that address conflict in landscapes where both people and wildlife will have to coexist.
